# GhHB12, a HD-ZIP I Transcription Factor, Negatively Regulates the Cotton Resistance to *Verticillium dahliae*

**DOI:** 10.3390/ijms19123997

**Published:** 2018-12-12

**Authors:** Xin He, Tianyi Wang, Wan Zhu, Yujing Wang, Longfu Zhu

**Affiliations:** 1 Southern Regional Collaborative Innovation Center for Grain and Oil Crops in China, Hunan Agricultural University, Changsha 410128, China; hexinzhsh@126.com; 2 National Key Laboratory of Crop Genetic Improvement, Huazhong Agricultural University, Wuhan 430070, China; TianyiWang@webmail.hzau.edu.cn (T.W.); 15377580390@163.com (W.Z.); 13072766389@163.com (Y.W.)

**Keywords:** cotton, Gossypium hirsutum, HD-ZIP, JA, Verticillium dahliae

## Abstract

The homeodomain-leucine zipper (HD-ZIP) is a plant-specific transcription factor family that plays important roles in plant developmental processes in response to multiple stressors. We previously isolated a cotton HD-ZIP class I transcription factor gene, *GhHB12*, which is regulated by the circadian clock and photoperiodism. Furthermore, it regulates cotton architecture, phase transition, and photoperiod sensitivity. Here we report that *GhHB12* was induced by methyl jasmonate (MeJA) and *Verticillium dahliae* infection. Additionally, stress-responsive elements were found in the *GhHB12* promoter. Promoter fusion analysis showed that *GhHB12* was predominantly expressed in primary roots and that it was induced by mechanical damage. Overexpression of *GhHB12* increased susceptibility of the cotton plant to the fungal pathogens *Botrytis cinerea* and *V. dahliae*, which was coupled with suppression of the jasmonic acid (JA)-response genes *GhJAZ2* and *GhPR3*. Our results suggest that GhHB12, a cotton stress-responsive HD-ZIP I transcription factor, negatively regulates cotton resistance to *V. dahliae* by suppressing JA-response genes.

## 1. Introduction

Plants face various environmental challenges, including invasion by microorganisms, during every stage of their life cycle. Consequently, plants have evolved intricate mechanisms to perceive external stimulation and they have developed a complex signal transduction network to regulate their adaptation to biotic and abiotic stresses. Phytohormones, such as abscisic acid (ABA), salicylic acid (SA), jasmonic acid (JA), and ethylene (ET), primarily regulate the protective responses of plants against various stressors through synergistic and antagonistic actions [1,2,3,4].

Transcription factors play crucial roles in the phytohormone crosstalk network during plant abiotic and biotic stress responses [5,6]. For example, AtMYC2/JIN1/RD22BP1, a basic helix-loop-helix (bHLH) transcription factor, is not only important in the JA signaling pathway, but also a positive regulator of the ABA-dependent drought response [7,8]. The transcription factor WRKY70, a node of convergence for SA- and JA-mediated signals for plant defense, has recently been shown to be a negative regulator during the beginning of ABA-controlled stomatal closure [9,10].

Homeodomain-leucine zipper (HD-ZIP) transcription factors, which are unique to the plant kingdom, have a leucine zipper motif immediately downstream of the homeodomain (HD) and are grouped into four classes [11]. Functional data that is available on a subset of the class I and II genes show that a number of them are involved in developmental reprogramming in response to changes in environmental conditions. The expression of many HD-ZIP class I genes is dependent on ABA, abiotic stress, and light conditions [12]. ATHB7 and ATHB12, which are induced by water-limiting conditions and applications of ABA, act as positive transcriptional regulators of PP2C genes (*ABI1*, *ABI2*, *HAB1*, *HAB2*) and as negative regulators of ABA receptors (PYL5 and PYL8) [13]. Salt induction of *ATHB7* involves ABA or ABA-dependent components that function as systemic signals for *ATHB7* expression [14]. ATHB7 and ATHB12 confer a reduced growth phenotype that is typical of water-limiting conditions and that is associated with the inhibition of gibberellin acid (GA) biosynthesis [15,16]. In addition, ATHB7 and ATHB12 are important factors in the development of symptoms associated with beet severe curly top virus (BSCTV) [17]. ATHB6, induced by drought stress and ABA, is a negative regulator in the ABA signaling pathway by possibly interacting with ABI1. Although it shows a similar expression pattern to ATHB6, ATHB5 is a positive regulator of ABA responsiveness, mediating the inhibitory effect of ABA on growth during seedling establishment [18]. *Nicotiana attenuata* NaHD20 is induced in roots and leaves by ABA and water deficit, acts as a positive regulator for ABA biosynthesis in leaves during water stress, and plays a negative role in the timing of bolting and flower transitions [19]. Plants overexpressing sunflower HaHB1 and *Arabidopsis* AtHB13 show a marked freezing, drought, and salinity tolerance through the induction of proteins that stabilize membranes [20]. Few reports show that HD-ZIP transcription factors regulate the plant defense responses. *Nicotiana benthamiana* NbHB1 is a JA-dependent positive regulator of pathogen-induced plant cell death [21]. HaHB4 acts as a positive regulator in JA biosynthesis and as a negative regulator of ET sensitivity and SA accumulation during biotic stress and wounding responses [22].

Globally, cotton (*Gossypium* spp.) is one of the most economically important crops and it is used as a source of natural fiber. However, the yield and quality of cotton fiber is restricted by many unfavorable environmental conditions. Verticillium wilt in cotton plants is a devastating vascular disease caused by the soil-borne hemi-biotrophic fungal pathogen *V. dahliae* [23,24]. Previous analyses have identified that the JA, SA, ET, and BR signaling as well as lignin play important roles in the cotton response to *V. dahliae* [25,26,27,28]. Several kinds of transcriptional factors, such as GbERF1, GbWRKY1, GhATAF1, GhMYB108, HDTF1, and GhbHLH171, were shown to be functionally related to Verticillium wilt resistance [25,29,30,31,32]; however, no cotton HD-ZIP transcription factors have been fully characterized in the response of cotton to *V. dahliae*.

Previous studies have shown that a cotton HD-ZIP class I protein, GhHB12 (cotton EST accession: DW511649), is strongly induced by ABA and salt stress, whereas it is slightly induced by cold stress [33]. Moreover, it regulates cotton architecture, phase transition, and photoperiod sensitivity [34]. In this study, we show that GhHB12 participates in the regulation of fungal pathogen responses in cotton. The expression of *GhHB12* was induced by methyl jasmonate (MeJA) and *V. dahliae* infection. The overexpression of *GhHB12* in cotton resulted in an increased susceptibility to fungal pathogens *B. cinerea* and *V. dahliae*. In contrast, specific silencing of *GhHB12* through RNA-interference increased the tolerance to fungal pathogens. Expression analysis showed that JA-response genes (*GhJAZ2* and *GhPR3*) were repressed in *GhHB12*-overexpressing plants during *V. dahliae* responses. Our results suggest that GhHB12 is a negative regulator in JA signaling during *V. dahliae* responses.

## 2. Results

### 2.1. GhHB12 is Induced by MeJA and *V. dahliae* Infection

A previous study has shown that the *GhHB12* cDNA is 1084 bp in length and encodes a polypeptide of 239 amino acid residues (see the Supplementary File). Results of alignment analysis showed that GhHB12 contained a homeobox domain that was homologous to AtHB12 (AGI: At1G01720) with 53% similarity, and thus was named GhHB12 [34]. To identify the gene structure of *GhHB12*, gene-specific primers were used to amplify *GhHB12* from the genomic DNA. Results of the comparison between cDNA and genomic DNA sequences revealed that the *GhHB12* gene consisted of two exons and one intron (Figure S1A), which were located on the chromosome Chr11-A (Gh_A11g0906) and Ghr11-D (Gh_D11g1052). Gh_A11g0906 was used to transform the cotton. Sequence analysis showed that Gh_A11g0906 and Gh_D11g1052 have only 6 single nucleotide polymorphism (SNP) at nucleotide level and 2 different amino acid residues at protein level. There are 23 SNP and 6 deletions between the promoters of Gh_A11g0906 and Gh_D11g1052 (see the Supplementary File).

**Figure 1:**
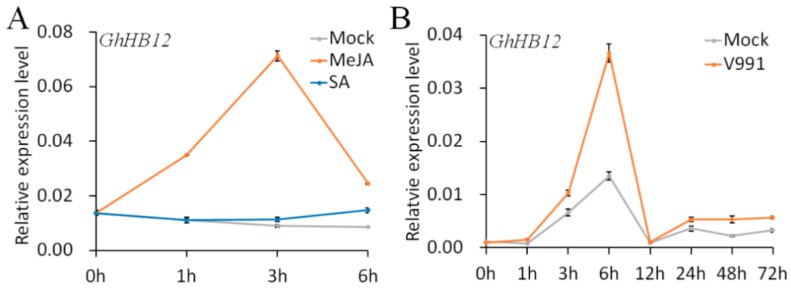
The expression patterns of *GhHB12* in response to (**A**) methyl jasmonate (MeJA) and salicylic acid (SA) and (**B**) *V. dahliae* infection. Total RNAs were extracted from roots of ”YZ1” seedlings at the indicated time points after treatments. The *GhUBQ7* gene was used as the endogenous reference gene. The data represent the mean ± SD of three technical replicates.

Our previous study showed that the expression of *GhHB12* (DW511497) was significantly increased by ABA and salt treatment [33]. MeJA, as an important phytohormone involved in the plant immune system, could quickly upregulate transcription of *GhHB12* (Figure 1A). However, *GhHB12* transcription did not respond to SA (Figure 1A). Moreover, *GhHB12* was also upregulated by *V. dahliae* (Figure 1B), a major fungal pathogen of cotton in China. These results indicated that *GhHB12* was involved in the response not only to abiotic stress, but also to biotic stress, possibly by regulating phytohormone signaling networks.

**Figure 2:**
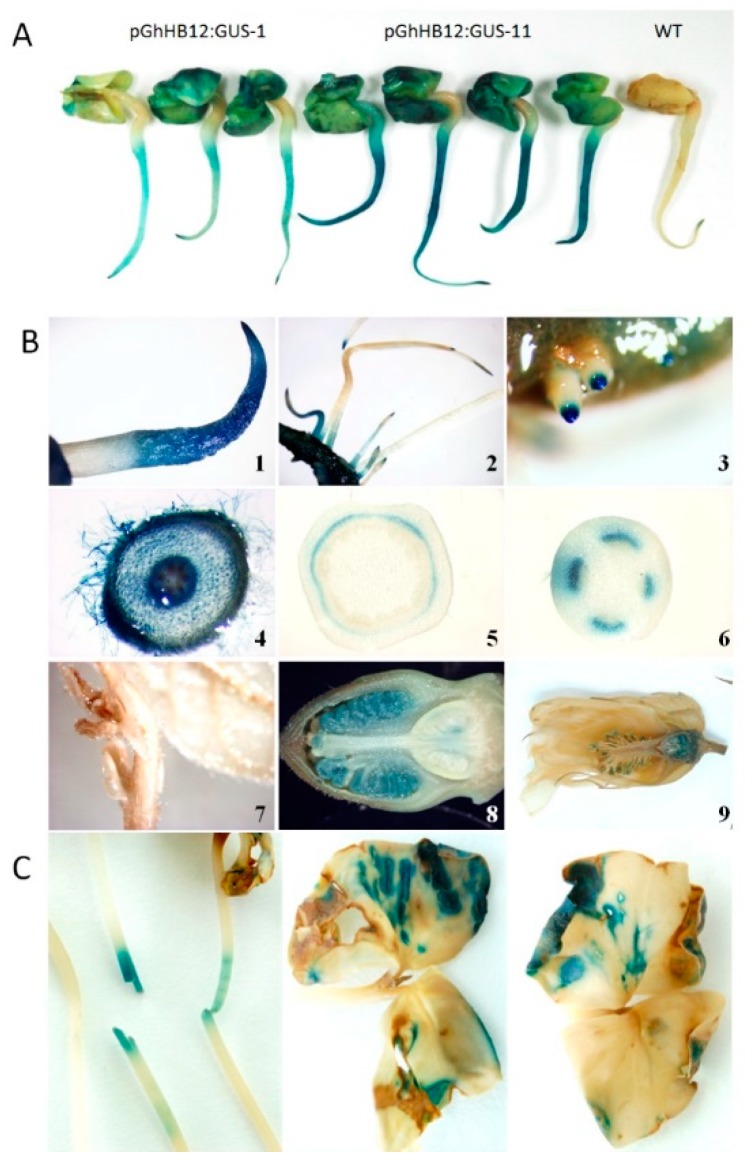
Histochemical localization of β-glucuronidase (GUS) activity in *pGhHB12:GUS* cotton plants. (**A**) *GhHB12* promoter drives GUS expression in the cotyledons and primary roots of the 3-day-old seedlings. (**B**) *GhHB12* promoter preferentially drives GUS expression in cotton primary roots, tips of lateral roots, vascular tissues, and floral organ. GUS staining in the (**1**) primary roots and (**4**) root hairs of the 3-day-old seedlings, (**2**,**3**) lateral roots of the 10-day-old seedlings, (**5**) section of stem, (**6**) petiole, (**7**) shoot apices and young leaves of 30-day-old plants, a longitudinal section of (**8**) buds and (**9**) flowers. (**C**) Wounding stress regulation of *GhHB12* promoters. GUS activity was detected in the mechanically damaged hypocotyls and cotyledons of the 10-day-old seedlings.

To determine if *GhHB12* upregulation by multiple stressors and phytohormones was controlled by its promoter, cotton was transformed with a construct bearing 905 base pairs (868 bp upstream and 37 bp downstream of the *GhHB12* start codon site) fused to the reporter gene β-glucuronidase (GUS). Histochemical analysis showed that GUS was expressed in cotton seedling cotyledons, primary roots, tips of lateral roots, vascular tissues, and the floral organ (Figure 2A,B). These results agree with the RT-PCR results of *GhHB12* expression in different cotton tissues and organs (Figure S1B). In addition, wounding stress induces GUS expression in cotton hypocotyls and cotyledons (Figure 2C).

**Figure 3:**
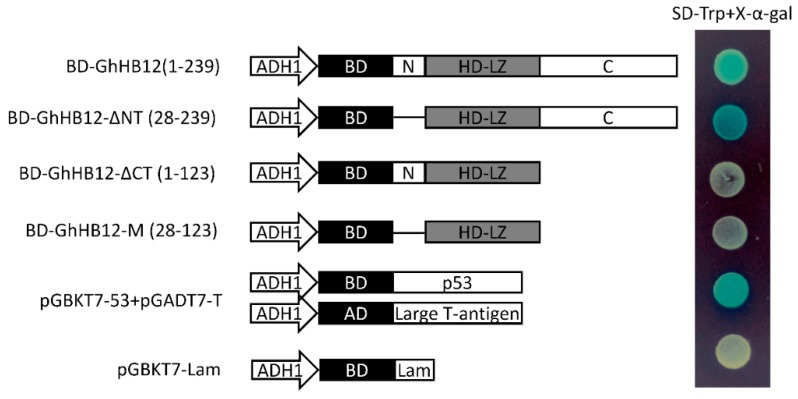
Transactivation analysis of the full-length GhHB12 protein and truncated GhHB12 proteins in yeast. The transformants were streaked on SD-Trp plus x-α-gal medium. pGBKT7-53+pGADT7-T was used as the positive controls, pGBKT7-Lam was used as the negative controls. BD: DNA binding domain of GAL4; AD: Activating domain of GAL4; ADH1: ADH1 promoter; N: N-terminal region; C: C-terminal region; HD-LZ: the HD and Zip domain; BD-GhHB12(1-239): Full-length GhHB12 (1-239 aa) fused to GAL4-BD; BD-GhHB12-ΔNT(28-239): GhHB12 without N-terminal region fused to GAL4-BD; BD-GhHB12-ΔCT(1-123): GhHB12 without C-terminal region fused to GAL4-BD; BD-GhHB12-M(28-123): GhHB12 without N-terminal and C-terminal region fused to GAL4-BD.

To test whether GhHB12 has any transcription activation properties, the full-length GhHB12 and different truncated GhHB12 sequences were fused to the DNA binding domain of GAL4 (GAL4-BD). The full-length GhHB12 (1-239 aa) fused to GAL4-BD produced strong transactivation activity, whereas the transactivation activity was abolished for both the N-terminal (N: 1-27 aa) and the HD and Zip (HD-LZ: 28-123 aa) domains of GhHB12 fused to GAL4-BD (Figure 3). These results indicated that the predicted C-terminus (C: 124-239 aa) was required for transcriptional activation in yeast. In addition, the transactivation activity of the truncated GhHB12 without the N-terminal region (GhHB12-ΔNT: 28-239 aa) was stronger than that of the full-length GhHB12 (Figure 3). Thus, this indicated that the N-terminal region of GhHB12 was a transcriptional repression region.

### 2.2. Reduced Fungal Pathogen Resistance of Transgenic Cotton Overexpressing *GhHB12*

**Figure 4:**
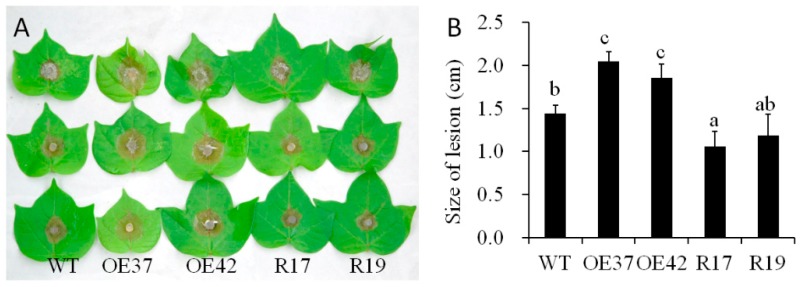
*GhHB12* negatively regulates the resistance to *B. cinerea* in cotton. (**A**) Phenotypes of *GhHB12* transgenic lines (overexpression lines: OE37 and OE42; RNAi lines: R17 and R19) and wild-type (WT) cotton leaves after inoculation with *B. cinerea* for three days. (**B**) The size of the lesions on the leaves indicated in (**A**). Error bars indicate the standard deviation of 7–22 lesions, and different letters indicate significant differences at *p* < 0.05 (Duncan’s multiple range test).

**Figure 5:**
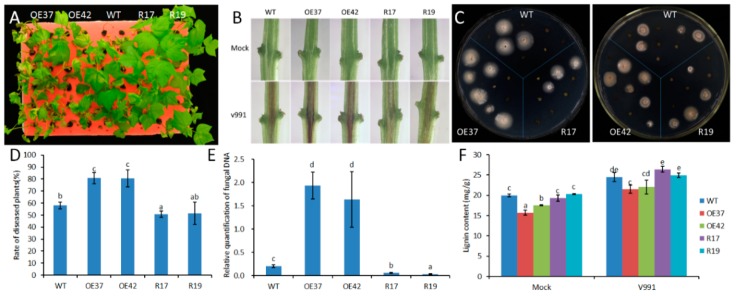
*GhHB12* negatively regulates the resistance to *V. dahliae* in cotton. (**A**) Cotton seedlings (wild-type: WT; overexpression lines: OE37 and OE42; RNAi lines: R17 and R19) 15 days after inoculation with *V. dahliae*. (**B**) Sections of cotton stems near the cotyledons after *V. dahliae* inoculation. The brown areas are diseased vascular bundles. (**C**) 15 days after *V. dahliae* inoculation, stem sections were plated, allowing fungal outgrowth as a measure for fungal colonization. (**D**) Rate of diseased plants 12 days after inoculation with *V. dahliae*. (**E**) qRT-PCR was used to analyze fungal colonization by comparing the *V. dahliae* internal transcribed spacer DNA levels (as a measure for fungal biomass) to the cotton *GhUBQ7* DNA levels 15 days post-inoculation. (**F**) Lignin content. The data represent the mean ± SE of three independent biological replications, and different letters indicate significant differences at *p* < 0.05 (Duncan’s multiple range test).

To evaluate the function of *GhHB12* during cotton resistance to fungal pathogens, overexpressed and RNA-interference (RNAi) constructs of *GhHB12* were assembled and transferred into cotton through *Agrobacterium*-mediated transformation. The transgenic cotton plants were confirmed by Southern blotting, RT-PCR, and RT-qPCR [34]. The two *GhHB12*-overexpressing cotton lines (OE37 and OE42) and the two *GhHB12*-RNAi cotton lines (R17 and R19) were used to analyze resistance to the fungal pathogens *B. cinerea* and *V. dahliae*. Leaves from *GhHB12* transgenic cotton lines and wild-type (WT) plants were inoculated with *B. cinerea* and the lesion sizes were measured three days after inoculation. Lesion sizes in the *GhHB12*-overexpressing lines were larger than those in wild-type plants, whereas they were smaller in the *GhHB12*-RNAi lines (Figure 4A,B). To analyze *V. dahliae* resistance responses in wild-type and *GhHB12* transgenic cotton plants, 2-week-old seedlings were inoculated with *V. dahliae* strain V991. The results showed that 15 days after inoculation, the *GhHB12*-overexpressing cotton lines presented more wilting and de-colored leaves than the wild-type plants, whereas *GhHB12*-RNAi cotton lines had fewer wilting and de-colored leaves (Figure 5A). These findings were further confirmed by the brown areas at the longitudinal sections of the cotton stems near the cotyledons node after *V. dahliae* inoculation (Figure 5B), the fungal recovery from the stem section of the inoculated plants (Figure 5C), the rate of diseased plants (Figure 5D), and the relative colonization of *V. dahliae* (Figure 5E).

Previous studies have demonstrated that lignin plays an important role in plant responses to fungal pathogens [27]. The lignin content was estimated in wild-type and *GhHB12* transgenic cotton lines. Inoculation of cotton seedlings with *V. dahliae* increased the total stem lignin content at 15 dpi; however, there was less lignin in the *GhHB12*-overexpression lines than in the wild-type. Meanwhile, slight or non-significant differences were observed between the *GHB12*-RNAi line and the wild-type (Figure 5F). These results demonstrated that *GhHB12* negatively regulated the resistance to *B. cinerea* and *V. dahliae* in cotton.

### 2.3. Changes in Expression of JA-Responsive Genes in *GhHB12* Transgenic Cotton Plants upon *V. dahliae* Infection

**Figure 6:**
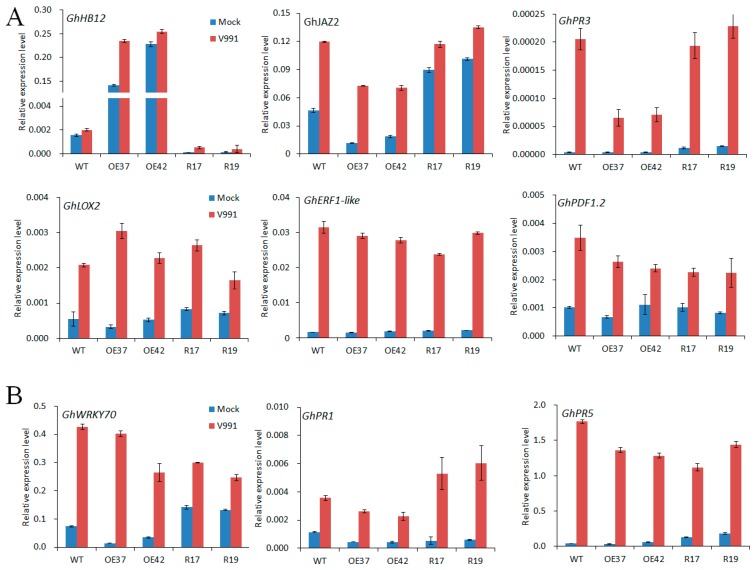
*GhHB12* reduced the expression of jasmonic acid (JA)-responsive defense maker genes (*GhJAZ2*, *GhPR3*) in cotton. qRT-PCR analysis of (**A**) JA-responsive defense maker genes and (**B**) SA-responsive defense maker genes in roots of wild-type (WT) and *GhHB12* transgenic cotton lines (overexpression lines: OE37 and OE42; RNAi lines: R17 and R19) after inoculation with or without *V. dahliae* V991. Total RNAs were extracted from roots of seedlings at the indicated time points (24 h) after 12 h inoculation. The *GhUBQ7* gene was used as the endogenous reference gene. The data represent the mean ± SD of three technical replicates.

JA and SA are important plant hormones that respond to fungal pathogens. To further validate the function of GhHB12 in JA and SA signaling responses during *V. dahliae* infection, the expression of JA- and SA-related defense genes in transgenic lines and wild-type plants was examined. Compared to the control treatment, the *GhJAZ2*, *GhPR3*, *GhLOX2*, *GhERF1*, and *GhPDF1.2* transcripts were increased in all plants after 24 h inoculation with strain V991, but fewer *GhJAZ2* and *GhPR3* transcripts were found in the *GhHB12*-overexpressing lines than in the wild-type and *GhHB12*-RNAi lines (Figure 6A). The *GhWRKY70*, *GhPR1*, and *GhPR5* transcripts were induced by infection with strain V991 similar to the JA-responsive genes, but no obvious change was found between the *GhHB12*-overexpressing lines and the wild-type (Figure 6B). Only the transcript level of *GhPR1* was upregulated in the *GhHB12*-RNAi plants in relation to the wild-type and *GhHB12*-overexpressing lines (Figure 6B). These results indicated that the overexpression of *GhHB12* repressed some JA-responsive genes in cotton.

## 3. Discussion

HD-ZIP transcription factors are known to play important roles during plant development and stress responses. The HD-ZIP I proteins are generally involved in responses related to abiotic stress, abscisic acid (ABA), blue light, de-etiolation, and embryogenesis [11]. ATHB7 and ATHB12, induced by drought stress and ABA, act as negative regulators of plant tolerance to abiotic stress by repressing ABA signaling [11]. Although it has been reported that ATHB7 and ATHB12 are important factors in beet severe curly top virus (BSCTV)-induced symptom development [17], the molecular mechanism is not well documented. In this study, we demonstrated that a cotton HD-ZIP I transcription factor, GhHB12, negatively regulates *GhJAZ2* and *GhPR3*, as well as the plant’s resistance to *V. dahliae*. Our previous study showed that GhHB12 promoted bushy architecture and delayed flowering in cotton [34]. Therefore, we can edit the two homologues of *GhHB12* in cotton using the CRISPR/Cas9 system to generate early-maturing and *V. dahliae*-tolerant cotton mutant lines.

Recent reports show that the JA signaling may play a positive role in the disease resistance of cotton [25,26,30,32,35] and in tomato [23] to *V. dahlia*. Furthermore, some reports have demonstrated that SA signaling also plays an important role in plant resistance to *V. dahliae* [28,36], even though SA is an antagonist of JA signaling during plant defense responses. In this study, we found that both the JA- and SA-response genes were induced in cotton roots after unimpaired root-dip inoculation with *V. dahliae* (Figure 6), which is consistent with the hemi-biotrophic lifestyle of *V. dahliae*. Though both the JA- and SA-response genes were induced by *V. dahliae* infection, only the expression of *GhJAZ2* and *GhPR3* genes in the *GhHB12*-overexpressing lines was consistently less than that in the wild-type and *GhHB12*-RNAi lines (Figure 6). Our previous reports showed that *GhJAZ2* and *GhPR3* were induced by MeJA [30,32]. Accordingly, the expression of *GhHB12* was induced by MeJA, *V. dahliae* infection, and mechanical damage, but it was not induced by SA (Figure 1 and Figure 2C). Taken together, these results suggest that *GhHB12* is involved in the responses to *V. dahliae* and JA signaling, but not SA signaling. There are few reports that show that HD-ZIP transcription factors are involved in plant defense responses. During biotic stress and wounding responses, HaHB4 is a positive regulator in JA biosynthesis, whereas it is a negative regulator in ET sensitivity and SA accumulation [22]. In this study, GhHB12 suppressed some JA-response genes, but did not activate SA signaling (Figure 6). Additionally, the JA biosynthesis gene (*GhLOX2*) and the JA-response maker gene (*GhPDF1.2*) were not repressed by GhHB12 (Figure 6A), suggesting that GhHB12 only represses some JA-response genes (*GhJAZ2* and *GhPR3*) but not the whole JA signaling pathway in cotton.

PR3 is a chitinase that hydrolyzes chitin, which is a major component of fungal cell walls [37]. Furthermore, plant chitinases have antifungal activity in vitro and can act synergistically with β-1, 3-glucanase to inhibit fungal growth, which enhances disease resistance [38,39]. Some reports show that the expression of chitinases in cotton, tomato, and strawberry improves plant resistance to *V. dahliae* [40,41,42,43]. Additionally, the silencing of three cotton chitinases impaired resistance against *V. dahliae* [44]. Our results illustrated that GhHB12 repressed the expression of *GhPR3* (Figure 6A), thereby supporting cotton resistance against *V. dahliae*.

Previous studies have demonstrated that lignin plays an important role in plant responses to fungal and insect attacks [27]. In this study, we found that GhHB12 decreased the lignin content in cotton stems with and without *V. dahliae* infection (Figure 5F). Gb/GhERF1-like genes directly promote the expression of *GhHCT1* involved in lignin synthesis in cotton, increasing the resistance of cotton to *V. dahliae* [29]. There is no significant difference in the expression of GhERF1-like genes between the wild-type and the *GhHB12* transgenic cotton plants (Figure 6A), suggesting that GhHB12 decreases lignin synthesis independently of GhERF1-like genes. Further analysis of the expression of lignin synthesis genes will expand our understanding of GhHB12 functions during cotton lignin synthesis in the response to *V. dahliae*.

GhHB12 is a repressor during the regulation of *GhPR3* and *GhJAZ2*. Its transactivation activity is strong (Figure 3), suggesting that GhHB12 may interact with some transcription repressors, subsequently suppressing some JA-response genes. Therefore, further characterization of protein interactions with GhHB12 will expand our understanding of the functions of GhHB12 in cotton defense responses.

## 4. Materials and Methods

### 4.1. Plant Materials, Growth Conditions, and Stress Treatments

The seedlings of upland cotton (*Gossypium hirsutum* L. cv. YZ1) were grown in Hoagland solution under 16 h light/8 h dark conditions for 2–3 weeks. The cotton seedling roots were infected with 1 × 10^7^ spore·mL^−1^ suspension of *V. dahliae* spores by unimpaired root-dip inoculation for 12 h, and then transplanted into distilled water. Control plants were inoculated with distilled water rather than spores but otherwise treated in the same way as the experimental plants. Roots were harvested at different time points (0 h, 1 h, 3 h, 6 h, 12 h, 24 h, 48 h, 72 h) after 12 h inoculation for RNA extraction. For treatments with MeJA and SA, the concentrations were applied at 100 µM and 1 mM in Hoagland solution, respectively [32]. The roots were harvested at different time points (0 h, 1 h, 3 h, 6 h) after treatment for later RNA isolation.

### 4.2. Expression Analysis

Total RNA from both control and stressed cotton tissues were isolated using a modified guanidine thiocyanate method [45]. For RT-qPCR analyses, 3 µg of total RNA/sample were used for cDNA biosynthesis with Superscript III reverse transcriptase (Invitrogen, San Diego, CA, USA). RT-qPCR was performed with SYBR green on the 7500 Real Time PCR System (ABI, Foster City, CA, USA). The relative changes were calculated with 2^−∆Ct^ and the cotton *GhUBQ7* gene (GeneBank: DQ116441) from *G. hirsutum* was amplified as the endogenous reference gene [46]. The relative transcript level was determined and normalized using the reference level and then averaged over the three technical replicates. Primers for the RT-qPCR (Supplemental Materials Table S1) were designed according to the cDNA sequences with Primer Premier 5 (http://www.premierbiosoft.com/crm/jsp/com/pbi/crm/clientside/ProductList.jsp).

### 4.3. Promoter Analysis and Histochemical Assay of GUS Activity

The promoter of *GhHB12* was cloned into the pGWB433 vector, which was then transferred into YZ1 by an *Agrobacterium*-mediated method [47]. Histochemical analysis of GUS was performed as described by Deng et al. [48]. Fresh tissue was collected from transgenic cotton plants and then immediately infiltrated with GUS staining solution at 37 °C for 6–12 h. The stained tissue was rinsed 3–5 times with 75% ethanol and then photographed under a microscope (Leica MZFLIII, Solms, Germany) or with a camera (Nikon, Tokyo, Japan).

### 4.4. Transcriptional Activation Activity of the *GhHB12* Protein

For the transactivation assay, the full open reading frame (ORF) and the different truncations of GhHB12 were obtained by PCR using the primers listed in Supplemental Table S1. The PCR products were fused in frame to the GAL4 DNA-binding domain in pGBKT7 through a recombination reaction. The fusion vectors were used to transform the yeast strain Y2HGold with the reporter gene TRP1, according to the manufacturer’s protocols. The transformed strains were streaked on plates of SD-Trp plus x-α-gal media.

### 4.5. Fungal Pathogen Cultivation and Inoculation

The *V. dahliae* strain V991 was incubated on PDA for one week and then inoculated into Czapek broth. The culture was then incubated on a shaker at 120 rpm at 25 °C for 3–4 days until the concentration of spores reached ~10^8^ spore·mL^−1^. The liquid suspension was adjusted 1 × 10^7^ spore·mL^−1^ with sterile distilled water for inoculation. The roots of the cotton seedlings grown under hydroponic conditions for three weeks were infected by unimpaired root-dip inoculation into the spore suspension (1 × 10^7^ spore·mL^−1^) for 12 h, and then transplanted into distilled water. Control plants (mock) were treated with distilled water. The severity of the disease symptoms on each cotton seedling was scored using a 0–4 rating scale as described previously [27]. RT-qPCR of the fungal colonization was performed by comparing the DNA levels between the *V. dahliae* internal transcribed spacer (ITS) (as a measure of fungal biomass) and the cotton *GhUBQ7* at 15 days post-inoculation in representative cotton stems sampled from above the cotyledons [49]. Stem sections sampled above the cotyledons were taken from plants after *V. dahliae* inoculation. They were then surface sterilized for 15 min in 70% ethanol, followed by 15 min in 10% hypochlorite and finally rinsed three times with sterile water. The sections were sliced and then transferred onto supplemented potato dextrose agar and incubated at 22 °C [50]. 

A *Botrytis cinerea* strain (stored at 4 °C) was transferred onto PDA medium and cultured for 5 days and then further incubated on fresh PDA medium for another 5 days at 25 °C. The disks of colonized agar were inoculated onto the excised leaves of 3-week-old plants at 25 °C, which were kept under a cover to maintain high humidity. Three days after inoculation, lesion sizes were measured.

### 4.6. Determination of Lignin Content

A representative sample of 100 mg of dry stem fine powder was extracted with 1.5 mL of 80% methanol overnight on a shaker 150 rpm at room temperature. The resulting residue was used for determination of lignin. The total lignin content was determined in triplicate from 100 mg of dry stem powder using the Klason method with modifications [51].

## 5. Conclusions

In this study, we identified a cotton homeodomain-leucine zipper transcription factor, GhHB12. This transcription factor is expressed in roots and vascular tissues, is induced by MeJA and *V. dahliae* infection, represses the expression of *GhPR3* and *GhJAZ2*, and negatively regulates resistance to the fungal pathogens *B. cinerea* and *V. dahliae* in cotton.

## Supplementary Materials

The following are available online at http://www.mdpi.com/1422-0067/19/12/3997/s1.

## References

[1] Atkinson N.J., Urwin P.E. (2012). The interaction of plant biotic and abiotic stresses: From genes to the field. J. Exp. Bot..

[2] Robert-Seilaniantz A., Grant M., Jones J.D. (2011). Hormone crosstalk in plant disease and defense: More than just jasmonate-salicylate antagonism. Annu. Rev. Phytopathol..

[3] Pieterse C.M., Leon-Reyes A., Van der Ent S., Van Wees S.C. (2009). Networking by small-molecule hormones in plant immunity. Nat. Chem. Biol..

[4] Pieterse C.M., Van der Does D., Zamioudis C., Leon-Reyes A., Van Wees S.C. (2012). Hormonal modulation of plant immunity. Annu. Rev. Cell Dev. Biol..

[5] Koornneef A., Pieterse C.M. (2008). Cross talk in defense signaling. Plant Physiol..

[6] Fujita M., Fujita Y., Noutoshi Y., Takahashi F., Narusaka Y., Yamaguchi-Shinozaki K., Shinozaki K. (2006). Crosstalk between abiotic and biotic stress responses: A current view from the points of convergence in the stress signaling networks. Curr. Opin. Plant Biol..

[7] Dombrecht B., Xue G.P., Sprague S.J., Kirkegaard J.A., Ross J.J., Reid J.B., Fitt G.P., Sewelam N., Schenk P.M., Manners J.M. (2007). MYC2 differentially modulates diverse jasmonate-dependent functions in Arabidopsis. Plant Cell.

[8] Abe H., Urao T., Ito T., Seki M., Shinozaki K., Yamaguchi-Shinozaki K. (2003). Arabidopsis AtMYC2 (bHLH) and AtMYB2 (MYB) function as transcriptional activators in abscisic acid signaling. Plant Cell.

[9] Li J., Brader G., Palva E.T. (2004). The WRKY70 transcription factor: A node of convergence for jasmonate-mediated and salicylate-mediated signals in plant defense. Plant Cell.

[10] Li J., Besseau S., Toronen P., Sipari N., Kollist H., Holm L., Palva E.T. (2013). Defense-related transcription factors WRKY70 and WRKY54 modulate osmotic stress tolerance by regulating stomatal aperture in Arabidopsis. New Phytol..

[11] Ariel F.D., Manavella P.A., Dezar C.A., Chan R.L. (2007). The true story of the HD-Zip family. Trends Plant Sci..

[12] Henriksson E., Olsson A.S.B., Johannesson H., Johansson H., Hanson J., Engstrom P., Soderman E. (2005). Homeodomain leucine zipper class I genes in Arabidopsis. Expression patterns and phylogenetic relationships. Plant Physiol..

[13] Valdes A.E., Overnas E., Johansson H., Rada-Iglesias A., Engstrom P. (2012). The homeodomain-leucine zipper (HD-Zip) class I transcription factors ATHB7 and ATHB12 modulate abscisic acid signalling by regulating protein phosphatase 2C and abscisic acid receptor gene activities. Plant Mol. Biol..

[14] Henriksson E., Henriksson K.N. (2005). Salt-stress signalling and the role of calcium in the regulation of the Arabidopsis ATHB7 gene. Plant Cell Environ..

[15] Olsson A.S., Engstrom P., Soderman E. (2004). The homeobox genes ATHB12 and ATHB7 encode potential regulators of growth in response to water deficit in Arabidopsis. Plant Mol. Biol..

[16] Son O., Hur Y.S., Kim Y.K., Lee H.J., Kim S., Kim M.R., Nam K.H., Lee M.S., Kim B.Y., Park J. (2010). ATHB12, an ABA-inducible homeodomain-leucine zipper (HD-Zip) protein of Arabidopsis, negatively regulates the growth of the inflorescence stem by decreasing the expression of a gibberellin 20-oxidase gene. Plant Cell Physiol..

[17] Park J., Lee H.J., Cheon C.I., Kim S.H., Hur Y.S., Auh C.K., Im K.H., Yun D.J., Lee S., Davis K.R. (2011). The Arabidopsis thaliana homeobox gene ATHB12 is involved in symptom development caused by geminivirus infection. PLoS ONE.

[18] Johannesson H., Wang Y., Hanson J., Engstrom P. (2003). The Arabidopsis thaliana homeobox gene ATHB5 is a potential regulator of abscisic acid responsiveness in developing seedlings. Plant Mol. Biol..

[19] Re D.A., Dezar C.A., Chan R.L., Baldwin I.T., Bonaventure G. (2011). Nicotiana attenuata NaHD20 plays a role in leaf ABA accumulation during water stress, benzylacetone emission from flowers, and the timing of bolting and flower transitions. J. Exp. Bot..

[20] Cabello J.V., Arce A.L., Chan R.L. (2012). The homologous HD-Zip I transcription factors HaHB1 and AtHB13 confer cold tolerance via the induction of pathogenesis-related and glucanase proteins. Plant J..

[21] Yoon J., Chung W.I., Choi D. (2009). NbHB1, Nicotiana benthamiana homeobox 1, is a jasmonic acid-dependent positive regulator of pathogen-induced plant cell death. New Phytol..

[22] Manavella P.A., Dezar C.A., Bonaventure G., Baldwin I.T., Chan R.L. (2008). HAHB4, a sunflower HD-Zip protein, integrates signals from the jasmonic acid and ethylene pathways during wounding and biotic stress responses. Plant J..

[23] Thaler J.S., Owen B., Higgins V.J. (2004). The role of the jasmonate response in plant susceptibility to diverse pathogens with a range of lifestyles. Plant Physiol..

[24] Klosterman S.J., Atallah Z.K., Vallad G.E., Subbarao K.V. (2009). Diversity, pathogenicity, and management of verticillium species. Annu. Rev. Phytopathol..

[25] Li C., He X., Luo X., Xu L., Liu L., Min L., Jin L., Zhu L., Zhang X. (2014). Cotton WRKY1 mediates the plant defense-to-development transition during infection of cotton by Verticillium dahliae by activating JASMONATE ZIM-DOMAIN1 expression. Plant Physiol..

[26] Gao W., Long L., Zhu L.F., Xu L., Gao W.H., Sun L.Q., Liu L.L., Zhang X.L. (2013). Proteomic and virus-induced gene silencing (VIGS) Analyses reveal that gossypol, brassinosteroids, and jasmonic acid contribute to the resistance of cotton to Verticillium dahliae. Mol. Cell. Proteom..

[27] Xu L., Zhu L.F., Tu L.L., Liu L.L., Yuan D.J., Jin L., Long L., Zhang X.L. (2011). Lignin metabolism has a central role in the resistance of cotton to the wilt fungus Verticillium dahliae as revealed by RNA-Seq-dependent transcriptional analysis and histochemistry. J. Exp. Bot..

[28] Liu T.L., Song T.Q., Zhang X., Yuan H.B., Su L.M., Li W.L., Xu J., Liu S.H., Chen L.L., Chen T.Z. (2014). Unconventionally secreted effectors of two filamentous pathogens target plant salicylate biosynthesis. Nat. Commun..

[29] Guo W., Jin L., Miao Y., He X., Hu Q., Guo K., Zhu L., Zhang X. (2016). An ethylene response-related factor, GbERF1-like, from Gossypium barbadense improves resistance to Verticillium dahliae via activating lignin synthesis. Plant Mol. Biol..

[30] He X., Zhu L., Xu L., Guo W., Zhang X. (2016). GhATAF1, a NAC transcription factor, confers abiotic and biotic stress responses by regulating phytohormonal signaling networks. Plant Cell Rep..

[31] Cheng H.Q., Han L.B., Yang C.L., Wu X.M., Zhong N.Q., Wu J.H., Wang F.X., Wang H.Y., Xia G.X. (2016). The cotton MYB108 forms a positive feedback regulation loop with CML11 and participates in the defense response against Verticillium dahliae infection. J. Exp. Bot..

[32] He X., Zhu L., Wassan G.M., Wang Y., Miao Y., Shaban M., Hu H., Sun H., Zhang X. (2018). GhJAZ2 attenuates cotton resistance to biotic stresses via the inhibition of the transcriptional activity of GhbHLH171. Mol. Plant Pathol..

[33] Zhu L.-F., He X., Yuan D.-J., Xu L., Xu L., Tu L.-L., Shen G.-X., Zhang H., Zhang X.-L. (2011). Genome-Wide Identification of Genes Responsive to ABA and Cold/Salt Stresses in Gossypium hirsutum by Data-Mining and Expression Pattern Analysis. Agric. Sci. China.

[34] He X., Wang T., Xu Z., Liu N., Wang L., Hu Q., Luo X., Zhang X., Zhu L. (2018). The cotton HD-Zip family transcription factor GhHB12 regulates flowering time and plant architecture via the GhMir157-GhSPL pathway. Commun. Biol..

[35] Xu L., Zhang W., He X., Liu M., Zhang K., Shaban M., Sun L., Zhu J., Luo Y., Yuan D. (2014). Functional characterization of cotton genes responsive to Verticillium dahliae through bioinformatics and reverse genetics strategies. J. Exp. Bot..

[36] Yan Z., Xingfen W., Wei R., Jun Y., Zhiying M. (2016). Island Cotton Enhanced Disease Susceptibility 1 Gene Encoding a Lipase-Like Protein Plays a Crucial Role in Response to Verticillium dahliae by Regulating the SA Level and H2O2 Accumulation. Front. Plant Sci..

[37] Boller T., Boller T., Boller T., Boller T., Boller T. (1985). Induction of hydrolases as a defense reaction against pathogens. Ucla Symp. Mol. Cell. Biol..

[38] Punja Z.K., Zhang Y.-Y. (1993). Plant Chitinases and Their Roles in Resistance to Fungal Diseases. J. Nematol..

[39] Cletus J., Balasubramanian V., Vashisht D., Sakthivel N. (2013). Transgenic expression of plant chitinases to enhance disease resistance. Biotechnol. Lett..

[40] Tohidfar M., Mohammadi M., Ghareyazie B. (2005). Agrobacterium-mediated transformation of cotton (Gossypium hirsutum) using a heterologous bean chitinase gene. Plant Cell Tissue Organ Cult..

[41] Tohidfar M., Hossaini R., Bashir N.S., Meisam T. (2012). Enhanced Resistance to Verticillium dahliae in Transgenic Cotton Expressing an Endochitinase Gene from Phaseolus vulgaris. Czech J. Genet. Plant.

[42] Tabaeizadeh Z., Agharbaoui Z., Harrak H., Poysa V. (1999). Transgenic tomato plants expressing a Lycopersicon chilense chitinase gene demonstrate improved resistance to Verticillium dahliae race 2. Plant Cell Rep..

[43] Chalavi V., Tabaeizadeh Z., Thibodeau P. (2003). Enhanced resistance to Verticillium dahliae in transgenic strawberry plants expressing a Lycopersicon chilense chitinase gene. J. Am. Soc. Hortic. Sci..

[44] Xu J., Xu X.Y., Tian L.L., Wang G.L., Zhang X.Y., Wang X.Y., Guo W.Z. (2016). Discovery and identification of candidate genes from the chitinase gene family for Verticillium dahliae resistance in cotton. Sci. Rep..

[45] Zhu L.F., Li-Li T.U., Zeng F.C., Liu D.Q., Zhang X.L. (2005). An Improved Simple Protocol for Isolation of High Quality RNA from Gossypium spp. Suitable for cDNA Library Construction. Acta Agron. Sin..

[46] Tu L., Zhang X., Liu D., Jin S., Cao J., Zhu L., Deng F., Tan J., Zhang C. (2007). Suitable internal control genes for qRT-PCR normalization in cotton fiber development and somatic embryogenesis. Chin. Sci. Bull..

[47] Jin S., Zhang X., Liang S., Nie Y., Guo X., Huang C. (2005). Factors affecting transformation efficiency of embryogenic callus of Upland cotton (Gossypium hirsutum) with Agrobacterium tumefaciens. Plant Cell Tissue Organ Cult..

[48] Deng F., Tu L., Tan J., Li Y., Nie Y., Zhang X. (2012). GbPDF1 is involved in cotton fiber initiation via the core cis-element HDZIP2ATATHB2. Plant Physiol..

[49] Ellendorff U., Fradin E.F., de Jonge R., Thomma B.P.H.J. (2009). RNA silencing is required for Arabidopsis defence against Verticillium wilt disease. J. Exp. Bot..

[50] Fradin E.F., Zhang Z., Juarez Ayala J.C., Castroverde C.D., Nazar R.N., Robb J., Liu C.M., Thomma B.P. (2009). Genetic dissection of Verticillium wilt resistance mediated by tomato Ve1. Plant Physiol..

[51] Rodrigues F.A., Jurick W., Datnoff L.E., Jones J.B., Rollins J.A. (2005). Silcon influences cytological and molecular events in compatible and incompatible rice-Magnaporthe grisea interactions. Physiol. Mol. Plant Pathol..

