# Supplementary material for: GhHB12, a HD-ZIP I Transcription Factor, Negatively Regulates the Cotton Resistance to Verticillium dahliae
Source: Int J Mol Sci. 2018 Dec 12;19(12):3997. doi: 10.3390/ijms19123997 (PMC6321269; doi:10.3390/ijms19123997)
Supplement: Supplementary file 1 [file ijms-19-03997-s001.zip › Supplementary/Supplementary files.docx]

**GhHB12, a HD-ZIP I transcription factor, negatively regulates the cotton resistance to *Verticillium dahliae***

Xin He^1, 2^, Tianyi Wang^2^, Wan Zhu^2^, Yujing Wang^2^, Longfu Zhu^2^*

1 Southern Regional Collaborative Innovation Center for Grain and Oil Crops in China, Hunan Agricultural University, Changsha, Hunan 410128, P. R. China

2 National Key Laboratory of Crop Genetic Improvement, Huazhong Agricultural University, Wuhan, Hubei 430070, P. R. China

*Corresponding author: [lfzhu@mail.hzau.edu.cn](mailto:lfzhu@mail.hzau.edu.cn)

National Key Laboratory of Crop Genetic Improvement, Huazhong Agricultural University, Wuhan, Hubei 430070, P. R. China

Tel: +86-27-8728-0510

Fax: +86-27-8728-0016

Supplementary file (The nucleotide sequences, amino acid sequences and promoter sequences of Gh_A11g0906 and Gh_D11g1052)

>Gh_A11G0906_NBI-AD1_v1.1|Name=Gh_A11G0906|organism=Gossypium hirsutum |type=mRNA| length =720bp

ATGTTAGATGGGGAAGAATATAGAGAGGAGATGGGTGAGCCTTTTTCCAGCGTGGTTCAAGTTACACCGACTAAAAAGAAGAAAAACAAGAACAAGAGGAGGTTCAGCGATGAACAAATCAAATCTCTGGAATTGATGTTTGAATCGGAAACCAGGCTTGAACCTCGAAAGAAGTTGCAGGTGGCTAAAGAGTTGGGTTTGCAGCCACGACAGGTTGCCATATGGTTTCAGAACAAGAGAGCCAGGTGGAAATCCAAGCAGCTTGAACGAGATTACACCATCCTACAAGCCAATTACGATCTTCTAGCTTCCAAGTACGAAAGTTTAAAGAGAGAAAAGCAGGCCTTACTCACTCAGTTGCAGAAGCTGAACGATTTGATTAAGAAGCCGAAAGAGGAAGAGCAGTGTTGCGGACAAGTTAACGGTATGAGGTGCAGTGAGGGAGCCTCAGATAAGGGAGAGACGACTGTGAAGTCTGATTCAGAAGGGCAGCTTAGTTTATCAATGGGAAGATCGGAACATGCTCTAGGAGCTTTATCGGATGATGATAGTGCCATAAGGACGGATTACTTCGGACTGGAAGAAGAGCCCAACCTTATGAGCATGGTGGATCCAGCTGACGGTTCTTTATCCTCTCCAGAAGATTGGCGTAGTTTAGACTCTGATGGTCTTTTTGATCAGTCCCCTTGTGGTTACCAATGGTGGGATTTTTGGTCTTGA

>Gh_D11G1052_NBI-AD1_v1.1|Name=Gh_D11G1052|organism=Gossypium hirsutum|type=mRNA|length =720bp

ATGTTAGATGGGGAAGAATATAGAGAGGAGATGGGTGAGCCTTTTTCCAGCGTGGTTCAAGTTACACCGACTAAAAAGAAGAAAAACAAGAACAAGAGGAGGTTCAGCGATGAACAAATCAAATCTCTGGAATTGATGTTTGAATCGGAAACCAGGCTTGAACCTCGAAAGAAGTTGCAGGTGGCTAAAGAGTTGGGTTTGCAGCCACGACAGGTTGCCATATGGTTTCAGAACAAGAGAGCCAGGTGGAAATCCAAGCAGCTTGAACGAGATTACACCATCCTACAAGCCAATTACGATCATCTAGCTTCCAAGTACGAAAGTTTAAAGAGAGAAAAGCAGGCCTTACTCGCTCAGTTGCAGAAGCTGAACGATTTGATTAAGAAGCCGAAAGAGGAAGAGCAGTGTTGCGGACAAGTTAACGGTATGAGGTGCAGTGAGGGAGCCTCAGATAAGGGAGAGACGACTGTGAAGTCTGATTCAGAAGGGCAGCTTAGTTTATCAATGGGAAGATCGGAACATGCACTAGGAGCCTTATCGGATGATGATAGTGCCATAAGGACGGATTACTTCGGACTGGAAGAAGAGCCCAACCTTATGAGCATGGTGGATCCAGCTGACGGTTCTTTATCCTCTCCAGAAGATTGGCGCAGTTTAGACTCTGACGGTCTTTTTGATCAGTCCCCTTGTGGTTACCAATGGTGGGATTTTTGGTCTTGA

>GhHB12 (Sequence of *GhHB12* clone used for overexpression vector in this study)

GCAAAATTCAGGGCAAAGATGTTAGATGGGGAAGAATATAGAGAGGAGATGGGTGAGCCTTTTTCCAGCGTGGTTCAAGTTACACCGACTAAAAAGAAGAAAAACAAGAACAAGAGGAGGTTCAGCGATGAACAAATCAAATCTCTGGAATTGATGTTTGAATCGGAAACCAGGCTTGAACCTCGAAAGAAGTTGCAGGTGGCTAAAGAGTTGGGTTTGCAGCCACGACAGGTTGCCATATGGTTTCAGAACAAGAGAGCCAGGTGGAAATCCAAGCAGCTTGAACGAGATTACACCATCCTACAAGCCAATTACGATCTTCTAGCTTCCAAGTACGAAAGTTTAAAGAGAGAAAAGCAGGCCTTACTCACTCAGTTGCAGAAGCTGAACGATTTGATTAAGAAGCCGAAAGAGGAAGAGCAGTGTTGCGGACAAGTTAACGGTATGAGGTGCAGTGAGGGAGCCTCAGATAAGGGAGAGACGACTGTGAAGTCTGATTCAGAAGGGCAGCTTAGTTTATCAATGGGAAGATCGGAACATGCTCTAGGAGCTTTATCGGATGATGATAGTGCCATAAGGACGGATTACTTCGGACTGGAAGAAGAGCCCAACCTTATGAGCATGGTGGATCCAGCTGACGGTTCTTTATCCTCTCCAGAAGATTGGCGTAGTTTAGACTCTGATGGTCTTTTTGATCAGTCCCCTTGTGGTTACCAATGGTGGGATTTTTGGTCTTGAAATAACCAAACAAAAACAAAACTATATAGAGGAAAATGTATGCAAATAATCTTTCCTTCTTTACACTGGGAGACATGGGG

>Gh_A11G0906

MLDGEEYREEMGEPFSSVVQVTPTKKKKNKNKRRFSDEQIKSLELMFESETRLEPRKKLQVAKELGLQPRQVAIWFQNKRARWKSKQLERDYTILQANYDLLASKYESLKREKQALLTQLQKLNDLIKKPKEEEQCCGQVNGMRCSEGASDKGETTVKSDSEGQLSLSMGRSEHALGALSDDDSAIRTDYFGLEEEPNLMSMVDPADGSLSSPEDWRSLDSDGLFDQSPCGYQWWDFWS

>Gh_D11G1052

MLDGEEYREEMGEPFSSVVQVTPTKKKKNKNKRRFSDEQIKSLELMFESETRLEPRKKLQVAKELGLQPRQVAIWFQNKRARWKSKQLERDYTILQANYDHLASKYESLKREKQALLAQLQKLNDLIKKPKEEEQCCGQVNGMRCSEGASDKGETTVKSDSEGQLSLSMGRSEHALGALSDDDSAIRTDYFGLEEEPNLMSMVDPADGSLSSPEDWRSLDSDGLFDQSPCGYQWWDFWS

>GhHB12 (The amino acid sequence of GhHB12 clone used for overexpression vector in this study)

MLDGEEYREEMGEPFSSVVQVTPTKKKKNKNKRRFSDEQIKSLELMFESETRLEPRKKLQVAKELGLQPRQVAIWFQNKRARWKSKQLERDYTILQANYDLLASKYESLKREKQALLTQLQKLNDLIKKPKEEEQCCGQVNGMRCSEGASDKGETTVKSDSEGQLSLSMGRSEHALGALSDDDSAIRTDYFGLEEEPNLMSMVDPADGSLSSPEDWRSLDSDGLFDQSPCGYQWWDFWS

>Promotor of Gh_A11G0906 (-868bp to +37bp)

AATCAAAGTTAAGTACGCTGTGACAGCGACGCCTTGGAGCCAATCTCCTGGCTGGAGCTGGGTTCTTTTTTATCAATGGCTTAGCCGAGGAACGAGGCTGAAAGATGGATGGATGGATGGCAAGTTTCTCCAAGAAGCATGGATATATATATATATAGTTGTAGTTGGCTTGGTTGTAAATGTAATGTCAGTATATGAAATTAAAAGAATGAATTTTGATTTTAAGTGGTGGAACGTGATTCATGCAGCCGACATTTTCTATATATTATAAAAACAAGACTTGTTGAAGTAGTGACGGTGATGATTCTGACAAATTACATTTTAATATTGGATTAACTATTAACACATGAAACATAGAATGGGAAGTCTGTAAAATGTGAAGTGATGCTTATACATGATTATTTCAAATTCAATTACATAAAAGTTAGCCAAGAAAGAGAACCACTTGGAGTTGATGCCGACATAAAACCTTTACTGCTTCTTTTTCTACCTTAAGGACTTACCCCTCGGATTAATGAAATATCTTAGTAGGATTTAATTAAAATTTTTAAGAGCAAAAAAATCTAGCACAAAAGATAGGAATAGAAATGAAAATTTTTATAAAGGCAAAAGAGGAAAAGCACTGTACGTTATCAGAGATCCCATAGAGTTTGGATATAAAGTAGGAATTAGCAGAATTTAGACATCCTATGACACCCAAACCCAAGCAGACGGATACTTACGGTTGTATATAAATACCAGCATACTCACAAGTACAAACGTCTAAAAACATTCCCTGTTTCTTCGATTCGTACTGCTACCTACTGGCTTGCCGGACCAACATTCGCGGCACTTAAAAATTTATTAGGAAGCAAAATTCAGGGCAAAGATGTTAGATGGGGAAGAATATAGAGAGGAGATGGGTG

> Promotor of Gh_D11G1052 (-832bp to +37bp)

AATCAAAGTTTAAGTACGCGGTGACAGCGACGCCTTGGAACCAATCTCCTGGCTGGAGCTGGGTTCTTTTTTATCAATGGCTTAGCCGAGGAAGGAGGATGAAAGATGGATGGATGGATGGCAAGTATCTCCAAGAAGCATGGATATATATATATATGTCAGTATATGAAATTAAAAGAATGAATTTTGATTTTAAGTGGTGGAACGTGATTCATGCAGCCGACATTTTCTATATTATAAGAACAAGACTTGTTGAAGTTGTGACGGTGATGATTCTGACAAATTATATTTTAATACTGGATTAACTATTAACACATGAAACATAGGATGGGAAGTCTGTAAAATGTGAAGTGATGCTTAAACATGATTATTTCAAATTCAATTACATAATAAGTTAGCCAAGAAAGAGAACCACTTGGAGTTGATGCCGACATAAAACCTTTACTGCTTCCTTTTCTACCTTAAGGACTTACCCCTCAATATCTTACTAGGATTTAATTAAAATTTTTAAGGGCAAAAAAATCTAGCACAAAAGATAGGAGTAGAAATGAAAATTTTTATAAAGGCAAAAGAGGAAAAGCACTGTACGTTATCAGAGATCCCATAGGGTTTGGATATAAAGTAGGAATTAGCAGAATTTAGACATCCTGTGAGACCCAAACCCAAGCAGATGGATACTTACGGTTGTATATAAATACCAGCATATTCACAAGTACAAACGTCTAAAAACATTCCCTGTTTCTTCGATTCGTACTGCTACCTACTGGCTTGCCGGACCAACATTCACAGCACTTACACAAAATTTTATTAGGAAGCAAAATCCAGGGCAAAGATGTTAGATGGGGAAGAATATAGAGAGGAGATGGGTG


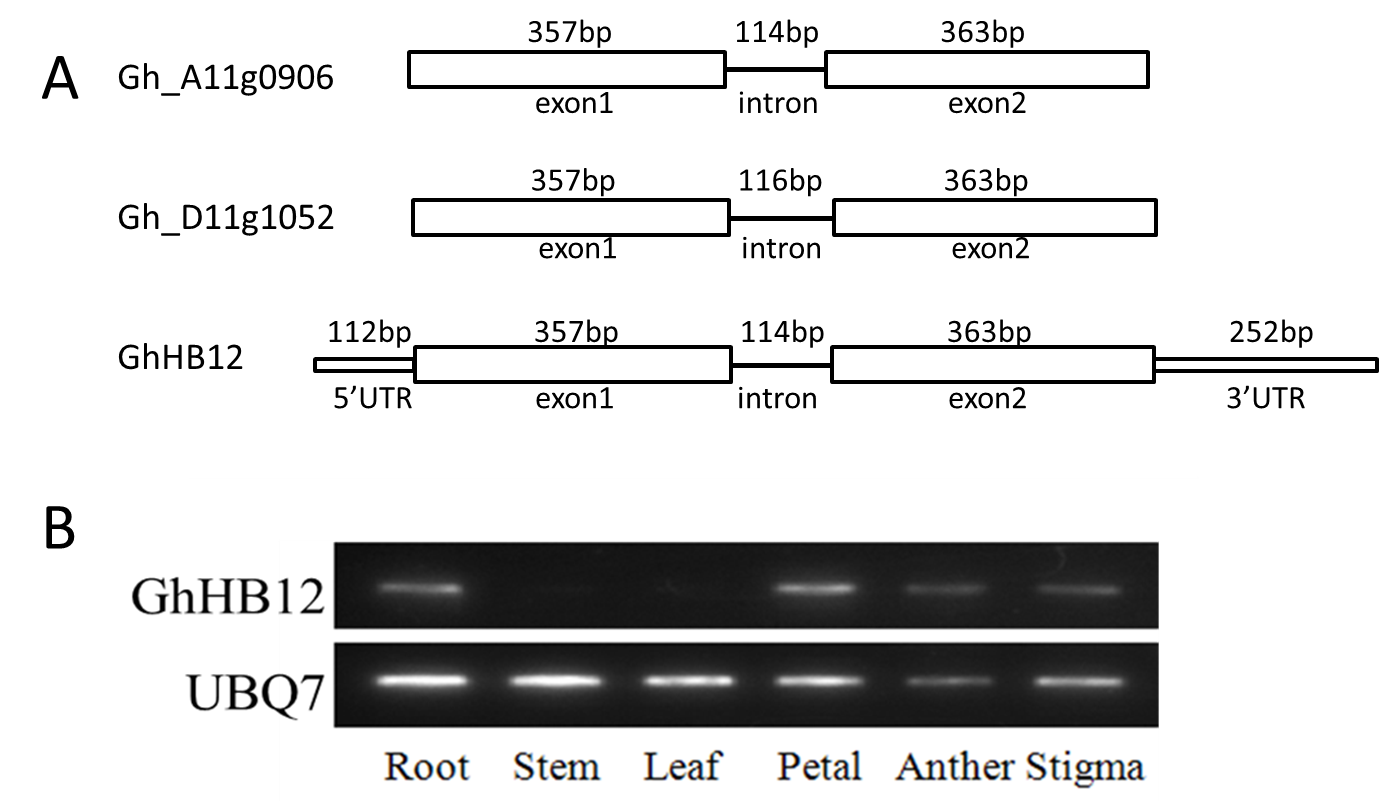


**Supplemental Figure 1. Gene structure and expression pattern of *GhHB12*.** A, Gene structures of *GhHB12,* *Gh_A11g0906* and *Gh_D11g1052*. B, Detection of the expression levels of *GhHB12* in the root, stem, leaf, petal, anther and stigma of upland cotton (*Gossypium hirsutum* L. YZ1) by RT-PCR.
